# Advances and future directions in *ROS1* fusion-positive lung cancer

**DOI:** 10.1093/oncolo/oyae205

**Published:** 2024-08-23

**Authors:** Mary C Boulanger, Jaime L Schneider, Jessica J Lin

**Affiliations:** Department of Medicine and Cancer Center, Massachusetts General Hospital, Boston, MA 02114, United States; Department of Medicine and Cancer Center, Massachusetts General Hospital, Boston, MA 02114, United States; Department of Medicine and Cancer Center, Massachusetts General Hospital, Boston, MA 02114, United States

**Keywords:** ROS1, non–small cell lung cancer, targeted therapy, tyrosine kinase inhibitor, drug resistance

## Abstract

*ROS1* gene fusions are an established oncogenic driver comprising 1%-2% of non–small cell lung cancer (NSCLC). Successful targeting of *ROS1* fusion oncoprotein with oral small-molecule tyrosine kinase inhibitors (TKIs) has revolutionized the treatment landscape of metastatic *ROS1* fusion-positive (ROS1+) NSCLC and transformed outcomes for patients. The preferred Food and Drug Administration-approved first-line therapies include crizotinib, entrectinib, and repotrectinib, and currently, selection amongst these options requires consideration of the systemic and CNS efficacy, tolerability, and access to therapy. Of note, resistance to ROS1 TKIs invariably develops, limiting the clinical benefit of these agents and leading to disease relapse. Progress in understanding the molecular mechanisms of resistance has enabled the development of numerous next-generation ROS1 TKIs, which achieve broader coverage of *ROS1* resistance mutations and superior CNS penetration than first-generation TKIs, as well as other therapeutic strategies to address TKI resistance. The approach to subsequent therapy depends on the pace and pattern of progressive disease on the initial ROS1 TKI and, if known, the mechanisms of TKI resistance. Herein, we describe a practical approach for the selection of initial and subsequent therapies for metastatic ROS1+ NSCLC based on these clinical considerations. Additionally, we explore the evolving evidence for the optimal treatment of earlier-stage, non–metastatic ROS1+ NSCLC, while, in parallel, highlighting future research directions with the goal of continuing to build on the tremendous progress in the management of ROS1+ NSCLC and ultimately improving the longevity and well-being of people living with this disease.

## Teaser

This article describes a practical approach for the selection of initial and subsequent therapies for metastatic ROS1+ non–small cell lung cancer, explores the evolving evidence for optimal treatment of early-stage disease, and highlights areas for future research.

**Implications for Practice**

Understanding of the biology of *ROS1* fusion-positive (ROS1+) non–small cell lung cancer (NSCLC) and treatment approaches has progressed immensely over the past decades, resulting in improved outcomes for patients. This review provides an overview of the evolving treatment landscape for ROS1+ NSCLC. The authors discuss the framework for the initial and subsequent treatment of patients with metastatic ROS1+ lung cancer and offer clinical considerations for the treatment of patients with earlier-stage, non–metastatic disease. Areas that warrant future research to continue to advance outcomes for patients are highlighted.

## Introduction

Since the discovery of *ROS1* gene fusions in non–small cell lung cancer (NSCLC) in 2007, our understanding of disease biology and therapeutic strategies has evolved remarkably, leading to improved patient outcomes.^1^  *ROS1* gene fusions are established drivers across diverse types of adult and pediatric cancers^2^ and result in the expression of a chimeric oncoprotein in which the tyrosine kinase domain of *ROS1* is fused to a non-native N-terminal binding partner.^3^ Aberrant expression and constitutive activation of *ROS1* lead to unchecked proliferation of tumor cells. The approval of effective oral small-molecule inhibitors of *ROS1* in *ROS1* fusion-positive (ROS1+) NSCLC has revolutionized its treatment paradigm.^4-6^ However, resistance to ROS1 tyrosine kinase inhibitors (TKIs) invariably occurs and causes disease relapse.^7^ Understanding the molecular mechanisms of resistance has been crucial in laying the groundwork for the development of next-generation ROS1 inhibitors and other novel strategies to tackle TKI-refractory disease.

Here, we provide an up-to-date overview of the arsenal of ROS1-targeted therapies that are approved or in clinical development. We discuss the approach to selecting agents for initial and subsequent treatment of patients with advanced ROS1+ lung cancer, and additionally provide a framework for treatment of patients with earlier-stage disease, highlighting future directions for research to facilitate continued progress in this field.

## Diagnosis of *ROS1* fusion-positive NSCLC

### Clinicopathologic features and epidemiology

*ROS1* fusions occur in 1%-2% of patients with NSCLC, accounting for approximately 18 500-37 000 newly diagnosed patients globally each year.^8-10^ In NSCLC, *ROS1* fusions are associated with adenocarcinoma histology, younger age at diagnosis, and a history of never or light smoking.^8,11^ Of note, *ROS1* fusions rarely overlap with other oncogenic driver alterations in de novo disease.^12^ Most patients (85%) with ROS1+ NSCLC present with stage IV cancer and 20%-40% have brain metastases at initial diagnosis.^13,14^

### Detection of ROS1 fusions and indications for testing

Over 30 *ROS1* fusion partner genes have been identified in NSCLC, most commonly *CD74*, *EZR*, *SCD4*, and *SLC34A2.*^1,4,15^ Broad molecular testing, including for *ROS1* fusions, is recommended by multiple guidelines including the National Comprehensive Cancer Network (NCCN) and European Society for Medical Oncology (ESMO) guidelines for patients with advanced lung adenocarcinoma, large cell, and NSCLC not otherwise specified, and should also be considered for those with advanced squamous cell carcinoma.^16,17^

Various methodologies are used to detect *ROS1* fusions, including fluorescent in situ hybridization (FISH), immunohistochemistry (IHC), real-time reverse transcription polymerase chain reaction (RT-PCR), and next-generation sequencing (NGS), each associated with advantages and limitations. Break-apart FISH was used in the early studies of ROS1+ NSCLC^8,12,18^ but can be technically challenging and lead to false-negative and false-positive results.^12^ While IHC is a faster method requiring minimal tissue, interpretation can also be challenging due to variations in ROS1 staining patterns and background ROS1 staining.^19^ Given the potential for false-positives, a positive ROS1 IHC result requires confirmatory testing with an orthogonal method.^12^ As the list of validated biomarkers in NSCLC continues to grow, NGS is a preferred method that enables probing for a broad panel of known oncogenes and can detect fusion partners, whether known or novel.^16,17^ However, NGS requires more tissue and has a slower turnaround time, and therefore, in circumstances where tissue is limited, FISH or RT-PCR may serve as alternatives. Liquid biopsy offers a noninvasive approach for biomarker testing, especially when tissue biopsy is not feasible or yields insufficient sample.^20^ On the other hand, tissue NGS has a higher sensitivity than plasma NGS, with sensitivity of the latter dependent on the overall burden of disease and shedding of circulating tumor DNA (ctDNA).^20^ Thus, it is essential to recognize that liquid biopsy can be nondiagnostic, and to consider rebiopsy to obtain adequate tissue for molecular testing when warranted.

## Overview of ROS1 Inhibitors

**Figure 1.**
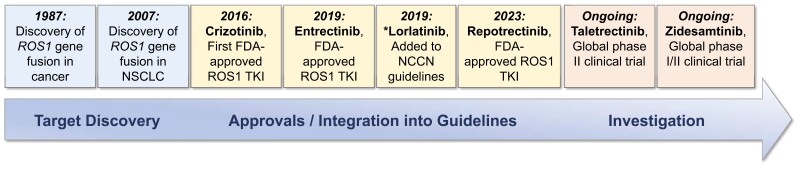
Timeline of advances in *ROS1* fusion-positive non–small cell lung cancer (NSCLC), including the discovery of *ROS1* gene fusions, approval of ROS1 tyrosine kinase inhibitors, and development of investigational ROS1 inhibitors. Crizotinib and entrectinib are also approved by the European Medicines Agency (EMA) and globally. *Lorlatinib is not approved by the FDA for the treatment of patients with metastatic *ROS1* fusion-positive NSCLC but is recommended as a subsequent treatment option by the NCCN guidelines and included in the ESMO guidelines.

In 2016, crizotinib became the first Food and Drug Administration (FDA)-approved targeted therapy for ROS1+ NSCLC and a harbinger of the development of additional, including next-generation, ROS1 TKIs (Figure 1).^21^ ROS1 shares 49% sequence homology to anaplastic lymphoma kinase (ALK) in the kinase domain (and 77% identity at the ATP-binding site), and this has proven clinically meaningful as certain—but not all (eg, alectinib)—ALK inhibitors also have anti-ROS1 activity.^3,22^ In this section, we review the key efficacy and safety data for ROS1 inhibitors, with additional investigational agents shown in Tables 1 and 2.

**Table 1.** First-line ROS1 inhibitor options.

Drug	Target kinases	Number of patients^a^	ORR	PFS (months)	Most common AEs^b^	Reference
Crizotinib^c^	ROS1, ALK, MET	53	72%	19.2	Vision disorder, diarrhea, nausea, edema, vomiting, elevated transaminases, constipation, and fatigue^d^	PROFILE 1001 Global phase I^4^ EUCROSS European phase II^23^ AcSé French phase II^24^ East Asia phase II^25^ METROS Italian phase II^26^
		30	70%	20.0		
		36	47.2%	5.5		
		127	71.7%	15.9		
		26	65%	22.8		
Entrectinib^c^	ROS1, TRK, ALK	168	68%	15.7	Dysgeusia, constipation, dizziness, diarrhea, weight gain, and fatigue	Integrated global phase I/II analysis: ALKA-372-001, STARTRK-1, and STARTRK-2^5,27^
Ceritinib	ROS1, ALK	30	67%	19.3	Diarrhea, nausea, anorexia, vomiting, cough, abdominal pain, musculoskeletal pain, fatigue, and dyspnea	Korean phase II^28^
Brigatinib	ROS1, ALK	28	67.9%	9.3	Diarrhea, increased CPK, nausea, cough, and hypertension	BAROSSA Japanese phase II^29^
Unecritinib	ROS1, ALK, MET	111	80.2%	16.5	Elevated AST, elevated ALT, vomiting, reduced neutrophil count, reduced leukocyte count, sinus bradycardia, diarrhea, elevated CPK, nausea, neurotoxicity (dysgeusia or dizziness), constipation, elevated LDH, elevated CK-MB, ocular disorders, elevated creatine, hypoproteinemia, anemia, and peripheral edema	Chinese phase II^30^
Lorlatinib	ROS1, ALK	21	62%	21.0	Hypercholesterolemia, hypertriglyceridemia, edema, peripheral neuropathy, cognitive effects, and weight gain	Global phase I/II^31^
		32	69%	35.8		Korean phase II^32^
Repotrectinib^c^	ROS1, TRK	71	79%	35.7	Dizziness, dysgeusia, constipation, anemia, paresthesia, dyspnea, elevated ALT, fatigue, ataxia, elevated AST, nausea, and muscular weakness	TRIDENT-1 Global phase I/II^6^
Taletrectinib	ROS1, TRK	106	90.6%	NR (33.2 months among 78 TKI-naïve patients in pooled phase I/II)	Diarrhea, elevated AST, elevated ALT, nausea, vomiting, anemia, neutrophil count decreased, WBC count decreased, bilirubin increased, dizziness, proteinuria, weight increase, creatinine increase, and prolonged QT	TRUST-I Chinese phase II^33,34^
		25	92.0%	Not reported		TRUST-II Global phase II^35^
Zidesamtinib	ROS1	Evaluation ongoing including in TKI-naïve patients				ARROS-1 Global phase I/II^36^

^a^The number of patients in the study or subgroup relevant to the efficacy evaluation in ROS1 TKI-naïve setting is shown.

^b^AEs reported in ≥20% of patients are shown.

^c^Indicated ROS1 TKIs are approved by the FDA for the treatment of metastatic *ROS1* fusion-positive NSCLC and included in the NCCN guidelines as preferred first-line options.

^d^AEs based on frequencies reported in the PROFILE1001 trial are shown.

Abbreviations: ORR, objective response rate; PFS, progression-free survival; AEs, adverse events; TKI, tyrosine kinase inhibitor; NR, not reached; AST, aspartate aminotransferase; ALT, alanine aminotransferase; CPK, creatine phosphokinase; LDH, lactate dehydrogenase; WBC, white blood cell.

**Table 2.** Second- or later-line ROS1 inhibitor options.

Drug	Number of patients^a^	ORR	PFS (months)	ORR in patients with known baseline *ROS1* G2032R	Reference
Lorlatinib	40 (prior crizotinib)	35%	8.5	0% (0/6)	Global phase I/II^31^
Repotrectinib^b^	56 (one previous TKI, no chemotherapy)	38%	9.0	59% (10/17)	TRIDENT-1 Global phase I/II^6^
Taletrectinib^c^	67 (prior crizotinib)	51.5%	7.6	66.7% (8/12)	TRUST-I Chinese phase II^33,34^
	21 (TKI-pretreated)	57.1%	Not reported	Not reported	TRUST-II Global phase II^35^
Zidesamtinib^c^	21 (TKI-pretreated)	48%^d^	Not reported	78% (7/9)	ARROS-1 Global phase I/II^36^

^a^The number of patients in the study or subgroup relevant to the efficacy evaluation in ROS1 TKI-pretreated setting is shown.

^b^Indicated ROS1 TKI is approved by the FDA for the treatment of metastatic *ROS1* fusion-positive NSCLC.

^c^Indicated ROS1 TKIs are being investigated in ongoing global phase II trials (NCT04919811 [TRUST-II] for taletrectinib and NCT05118789 [ARROS-1] for zidesamtinib).

^d^In the preliminary analysis from the ARROS-1 phase I portion, the ORR was 53% (9/17) among patients who received 2 or more prior ROS1 TKIs and 1 or more lines of prior chemotherapy, and 50% (9/18) among patients who received prior lorlatinib or repotrectinib.

Abbreviations: ORR, objective response rate; PFS, progression-free survival; TKI, tyrosine kinase inhibitor.

### Crizotinib

Crizotinib is a multikinase inhibitor (MKI) with activity against ROS1, ALK, and mesenchymal-epidermal transition (MET).^4,37^ The FDA approval of crizotinib for the treatment of patients with advanced ROS1*+* NSCLC was based on the results from a multicenter phase I trial PROFILE 1001, which demonstrated an objective response rate (ORR) of 72% and median progression-free survival (mPFS) of 19.2 months in this patient population.^4,21^ The most frequent adverse events (AEs) include visual impairment, nausea, vomiting, diarrhea, constipation, edema, and elevated aminotransferase levels. In 2019, updated results from PROFILE 1001 demonstrated a mPFS of 19.3 months and median overall survival (mOS) of 51.4 months with no new safety signals, confirming its efficacy and safety.^38^ Of note, other phase I/II studies of crizotinib in ROS1*+* NSCLC (ie, EUCROSS, AcSé, East Asia phase II, and METROS) have demonstrated variable PFS with median ranging from 5.5 to 22.8 months but with consistently high ORR’s ranging from 65% to 72%.^23-26^ A major limitation of crizotinib is the marginal penetration of the blood-brain barrier, resulting in limited CNS activity and frequent CNS disease relapses.^14^

### Entrectinib

Entrectinib, a ROS1, ALK, and TRK inhibitor, was approved by the FDA in 2019 for advanced ROS1+ NSCLC.^39^ An integrated analysis of ALKA-372-001, STARTRK-1, and STARTRK-2 trials demonstrated an ORR of 68%, mPFS of 15.7 months and mOS of 47.8 months.^5^ The most frequent entrectinib-associated AEs include dysgeusia, dizziness, and constipation. An important distinction from crizotinib is that entrectinib has improved CNS penetration and activity. The intracranial ORR among 25 patients with measurable baseline CNS metastases in the long-term integrated analysis was 80% with a median intracranial PFS of 8.8 months (Table 3).^27^

**Table 3.** CNS efficacy of ROS1 inhibitors.

Drug	Number of patients (with baseline CNS metastases)	Intracranial ORR^a^	Intracranial PFS (months)^b^	Reference
Crizotinib	N.A.	Not assessed	Not assessed Not assessed	PROFILE 1001 Global phase I^4^ EUCROSS European phase II^23^ AcSé French phase II^24^ East Asia phase II^25^
	6	33.3%		METROS Italian phase II^26^
Entrectinib	25	80%	8.8	Integrated global phase I/II analysis: ALKA-372-001, STARTRK-1, and STARTRK-2^27^
Ceritinib	8	25%	Not assessed	Korean phase II^28^
Unecritinib	11	72.7%	10.1	Chinese phase II^30^
Lorlatinib	11 (ROS1 TKI-naïve) 24 (crizotinib-pretreated)	64% 50%	Not reached Not reached	Global phase I/II Study^31^
	15 (crizotinib-pretreated)	87%	38.8 months	MGH/DFCI phase II trial^40^
Repotrectinib	9 (ROS1 TKI-naïve)^c^ 13 (1 prior ROS1 TKI, no prior chemotherapy)^c^	89% 38%	35.7 months^d^ Not reached^d^	TRIDENT-1 Global phase I/II^6^
Taletrectinib	8 (TKI-naïve) 15 (TKI-pretreated)	87.5% 73.3%	Not reported	TRUST-I Chinese phase II^33,34^
	5 (TKI-naïve) 8 (TKI-pretreated)	80% 62.5%	Not reported	TRUST-II Global phase II^35^
Zidesamtinib	Evaluation ongoing—intracranial responses reported			ARROS-1 Global phase I/II^36^

^a^Across trials, the intracranial ORR was measured using distinct criteria (eg, RANO-BM and RECIST) and in distinct populations of patients (eg, those with measurable CNS lesions only, or those with measurable and nonmeasurable CNS disease).

^b^The intracranial PFS in patients with known baseline CNS metastases is shown where available.

^c^Number of patients with baseline measurable CNS metastases is indicated.

^d^The median intracranial PFS in 17 TKI-naïve and 26 TKI-pretreated patients with any (measurable and nonmeasurable) baseline CNS metastases is shown.

Abbreviations: CNS, central nervous system; ORR, objective response rate; PFS, progression-free survival; N.A., not applicable; TKI, tyrosine kinase inhibitor; MGH, Massachusetts General Hospital; DFCI, Dana-Farber Cancer Institute.

### Lorlatinib

Lorlatinib is a potent and CNS-penetrant ALK and ROS1 inhibitor evaluated in a phase I/II clinical trial among patients with metastatic ROS1*+* NSCLC (30% TKI-naïve and 70% TKI-pretreated).^31^ Among TKI-naïve patients, the ORR was 62% and mPFS 21 months. Among crizotinib-pretreated patients, the ORR was 35% and mPFS 8.5 months. The most common AEs included hypercholesterolemia, hypertriglyceridemia, edema, neuropathy, cognitive effects, mood effects, and weight increase. Consistent with the robust CNS penetration of this TKI, the intracranial ORR was 64% for TKI-naïve and 50% for crizotinib-pretreated patients with baseline brain metastases, respectively, with median duration of intracranial response not reached. While the FDA-approved indication of lorlatinib is for advanced ALK-positive (ALK+) NSCLC, the NCCN guidelines include lorlatinib as a treatment option for patients with ROS1+ NSCLC and progression on a prior TKI based on these phase I/II trial data.^17,41^

### Repotrectinib

Repotrectinib is a next-generation ROS1 and TRK inhibitor.^42^ In the registrational phase I/II clinical trial TRIDENT-1, repotrectinib showed an ORR of 79%, intracranial ORR of 89%, and a mPFS of 35.7 months among ROS1 TKI-naïve patients.^6^ Among patients who had received one previous ROS1 TKI and no chemotherapy, the ORR was 38% and the intracranial ORR was 38%, with a mPFS of 9.0 months and mOS of 25.1 months. The most frequent AEs included dizziness, dysgeusia, paresthesias, constipation, and anemia. Of note, neurological toxicities of repotrectinib (and entrectinib) are attributed to the inhibition of TRK, which is widely expressed and involved in the central and peripheral nervous systems.^2^ Based on TRIDENT-1, the FDA approved repotrectinib in 2023 for line-agnostic treatment for locally advanced or metastatic ROS1+ NSCLC.^43^

### Taletrectinib (DS-6051b)

Taletrectinib is an investigational next-generation ROS1 inhibitor.^33^ In the Chinese phase II trial TRUST-I, taletrectinib demonstrated an ORR of 90.6% among ROS1 TKI-naïve patients (mPFS not reached), and an ORR of 51.5% and mPFS of 7.6 months among crizotinib-pretreated patients, with an intracranial ORR of 87.5% among TKI-naïve patients and 73.3% among crizotinib-pretreated patients with measurable brain metastases.^33,34^ In a pooled analysis of the Chinese phase I/II studies, the mPFS among TKI-naïve patients was 33.2 months. The most common AEs included increased AST, increased ALT, and diarrhea. Taletrectinib is being evaluated in the global phase II trial TRUST-II (NCT04919811) with early preliminary data showing an ORR of 92.0% and 57.1% in ROS1 TKI-naïve and TKI-pretreated patients, respectively.^35,44,45^

### Zidesamtinib (NVL-520)

Zidesamtinib is an investigational next-generation, brain-penetrant TKI that is ROS1-selective with activity against *ROS1* resistance mutations.^46^ Of note, zidesamtinib was developed to avoid TRK inhibition and associated neurological toxicities.^46^ Preliminary data from the phase I dose escalation portion of the global ARROS-1 trial (NCT05118789) demonstrated favorable tolerability of zidesamtinib concordant with its highly ROS1-selective design, with an ORR of 48% in heavily TKI-pretreated patients and documented CNS responses in patients with baseline brain metastases.^36,47,48^ In February 2024, the FDA granted breakthrough therapy designation to zidesamtinib for treatment of patients with metastatic ROS1*+* NSCLC after at least 2 ROS1 TKIs.^48,49^ The ARROS-1 trial has now transitioned to phase II and will evaluate the efficacy and safety in both TKI-naïve and TKI-pretreated patients.

## Approach to selection of initial therapy for metastatic disease

**Figure 2.**
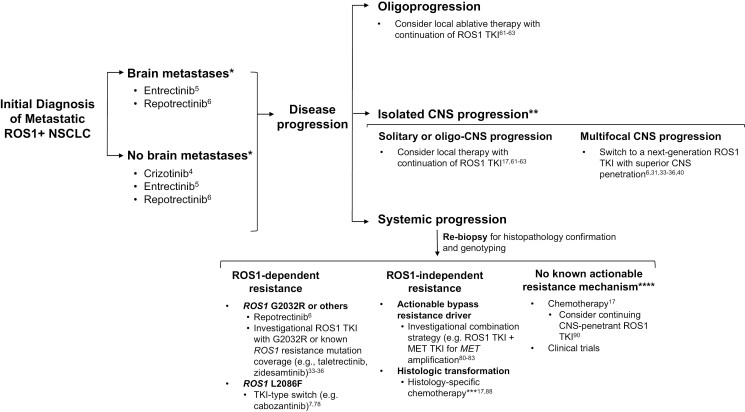
Approach to treatment of metastatic *ROS1* fusion-positive non–small cell lung cancer. Preferred FDA-approved first-line therapy options include crizotinib, entrectinib, or repotrectinib. *Investigational next-generation ROS1 inhibitors taletrectinib and zidesamtinib can also be considered in the first-line setting. At disease progression, the selection of subsequent treatment should be determined based on the pattern of progressive disease and (for addressing systemic progression with switch in systemic therapy) mechanism of drug resistance, if known through rebiopsy. **In addressing CNS progression on a ROS1 inhibitor, multidisciplinary evaluation to assess the optimal use of surgery, radiation, versus switch in systemic therapy is essential. ***Consider continuing ROS1 TKI for nontransformed clones. ****Consider also for polyclonal resistance or concurrent on- and off-target resistance. Abbreviations: TKI, tyrosine kinase inhibitor; ROS1+, *ROS1* fusion-positive; CNS, central nervous system.

With the emerging data on next-generation ROS1 TKIs, the first-line treatment landscape for metastatic ROS1+ NSCLC is evolving. Following the recent FDA approval of repotrectinib, the NCCN guidelines have been updated to include crizotinib, entrectinib, or repotrectinib as preferred first-line therapy for metastatic ROS1*+* NSCLC (Figure 2).^17,43^ Prospective trial data comparing each of these first-line options are not yet available; however, ongoing phase III trials are comparing crizotinib versus entrectinib (NCT04603807) and crizotinib versus repotrectinib (NCT06140836; TRIDENT-3).^50,51^

While awaiting the results of these direct comparison studies, the selection of first-line therapy requires weighing several factors including the systemic and CNS efficacy, tolerability, and access (eg, regulatory approval and drug reimbursement). Given the poor CNS penetration as a limitation of crizotinib, CNS-active TKIs entrectinib and repotrectinib are favored for patients with known brain metastases. Despite the lack of phase III trial results at this time, based on the available data, next-generation ROS1 TKIs such as repotrectinib and taletrectinib do appear to result in systemic PFS that is longer (median 33.2 to 35.7 months) than that historically reported for first-generation ROS1 TKIs crizotinib and entrectinib (median 15.7 to 19.2 months in global studies).^4-6,25,33^ This is reminiscent of what has been observed in randomized phase III trials of first- versus next-generation TKIs in ALK+ and *EGFR*-mutated NSCLC.^52-59^ Overall, next-generation ALK/EGFR TKIs demonstrated a clinically meaningful increase in mPFS compared to first-generation agents, resulting in their supplantation of earlier-generation TKIs as standard-of-care.^52-60^ Thus, across driver genotypes of NSCLC, the evolving treatment paradigm has reflected the shift of more potent, next-generation targeted therapies toward upfront rather than later-line use.

As additional next-generation ROS1 TKIs are evaluated in the first-line setting, the focus will be on whether there are key differences amongst the next-generation agents (eg, repotrectinib, taletrectinib, and zidesamtinib) in the systemic and CNS activity and tolerability. The initial treatment paradigm will continue to be refined as these questions are answered.

## Resistance to ROS1 inhibitors and selection of subsequent therapies

The approach to the selection of subsequent therapies should be determined based on the pace and pattern of progressive disease (PD) as well as on mechanisms of resistance to therapy, if known. Here, we provide a practical overview of treatment approaches in the setting of oligoprogression, isolated CNS progression, and systemic progression (Figure 2).

### Oligoprogression

Oligoprogression in therapy, defined as PD involving a limited number of sites with ongoing disease control elsewhere, is a well-recognized phenomenon.^61^ Several retrospective studies have demonstrated a role for local therapy (eg, radiation, surgery, and ablation) to address the oligo-PD in patients in *EGFR*-mutated or fusion oncogene-driven NSCLC on targeted therapy.^61,62^ For example, in one study that evaluated 61 patients with advanced *ALK* (*n* = 37), *ROS1* (*n* = 12), or *RET* (*n* = 12) fusion-positive lung adenocarcinomas treated with genotype-matched TKI, the receipt of local therapy for solitary or oligo-PD yielded a median of 6.8 months from local therapy to subsequent progression and a median of 10 months from local therapy to next systemic therapy.^62^ A prospective phase II randomized trial (CURB) demonstrated that stereotactic body radiotherapy (SBRT) to treat oligoprogressive metastatic NSCLC resulted in a more than 4-fold PFS benefit.^63^ Collectively, these data support that for patients who develop solitary or oligo-PD while on a ROS1 inhibitor, local therapy to the progressive lesions can be considered and may enable extended duration on a given TKI.

### Isolated CNS progression

CNS-only progression is another frequently observed pattern of PD on a ROS1 inhibitor. Indeed, in half of patients treated with crizotinib, CNS represents the sole site of PD.^14^ In general, the optimal approach to addressing CNS-only PD varies depending on the (1) TKI being used, (2) the pattern and extent of CNS involvement, and (3) symptoms from CNS disease, and requires multidisciplinary evaluation. For limited CNS PD, local therapy with focal brain-directed radiotherapy or neurosurgical resection may be used, but for multifocal CNS PD, a switch in systemic therapy (such as to a ROS1 TKI with a higher level of CNS penetrance, if available) or whole brain radiotherapy are considered.

Studies performed to date have focused on addressing CNS relapse in patients treated with crizotinib. While entrectinib is a CNS-active TKI, the intracranial efficacy was modest in patients experiencing CNS-only progression on crizotinib with an intracranial ORR of 19% and median intracranial PFS of 4.5 months.^27^ In contrast, in a phase II trial, lorlatinib induced deep, durable intracranial responses in patients with CNS-only progression on crizotinib (12-week intracranial ORR of 87%, complete intracranial response rate of 60%, median intracranial PFS of 38.8 months).^40^ More recently, next-generation ROS1 TKIs repotrectinib, taletrectinib, and zidesamtinib have shown encouraging CNS activity (Table 3).^6,33,36^ Thus, for patients experiencing multifocal CNS PD on a first-generation ROS1 inhibitor, switching to a next-generation ROS1 TKI with known CNS penetrance is favored. Our understanding of the CNS efficacy of next-generation agents following CNS PD on entrectinib or another CNS-penetrant next-generation TKI needs to be better delineated to optimize TKI sequencing strategies.

### Systemic progression

**Figure 3.**
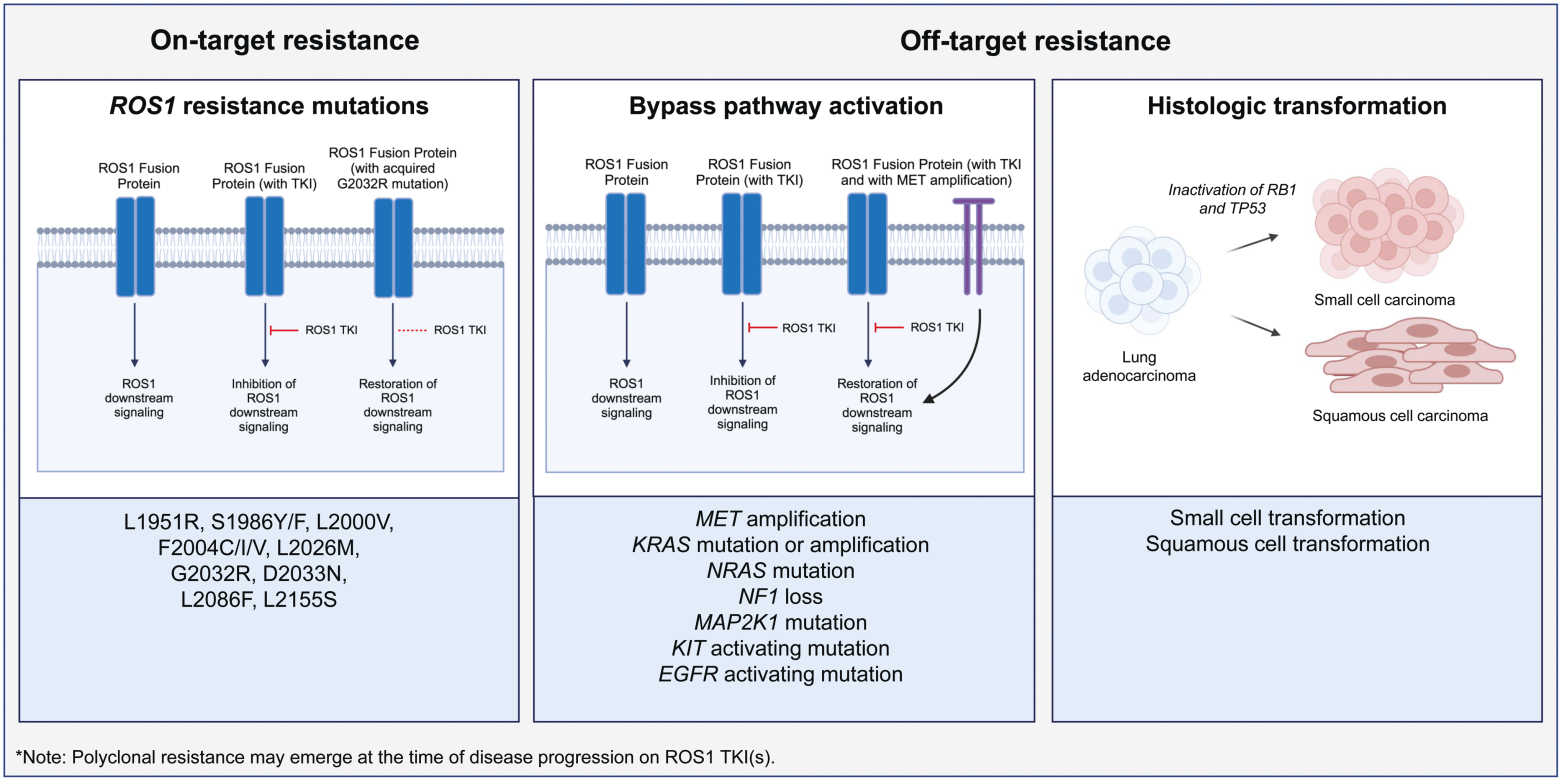
Mechanisms of resistance to ROS1 inhibitors. The mechanisms of resistance are broadly categorized as on-target (ie, *ROS1* resistance mutations^7,46,66,67^) and off-target resistance (ie, bypass pathway activation or histologic transformation^7,67-73^). Polyclonal resistance (or concurrent on- and/or off-target mechanisms of resistance) may also occur. Abbreviation: TKI, tyrosine kinase inhibitor. Created with BioRender.com.

Extrapolating from data in *EGFR*-mutated NSCLC, it is worth noting that for slow, asymptomatic progression, immediate change in therapy is not always required and patients may be able to continue a given ROS1 TKI while being cautiously monitored for change in the pace of PD or development of disease-related symptoms.^64,65^ In the face of significant systemic progression necessitating a switch in therapy, knowledge of the molecular mechanism of drug resistance (Figure 3) can inform subsequent treatment selection. Rebiopsy is ideal if safe and feasible, as it enables assessment for evidence of histologic transformation and genotyping for acquired alterations. Liquid biopsy with ctDNA NGS can also be considered, acknowledging that (1) sensitivity may be limited due to variable degrees of ctDNA shedding, (2) histologic transformations cannot be identified with liquid biopsies, and (3) detection of copy number changes may not always be reliable.^74^

#### On-target resistance

The emergence of on-target resistance is a recurrent theme across cancers in which the selective pressure of targeted therapy induces on-target genomic alterations to confer drug resistance. Various secondary point mutations in the ROS1 kinase domain at frequencies ranging from 8% to 46% have been described in ROS1+ NSCLC treated with ROS1 TKIs.^7,66^ The G2032R solvent front mutation is the most common *ROS1* resistance mutation (described in up to 41% of patients post-crizotinib and 32% post-lorlatinib), which causes steric interference to TKI binding.^7,66^ While crizotinib, entrectinib, and lorlatinib are not active against ROS1 G2032R, next-generation ROS1 TKIs (repotrectinib, taletrectinib, and zidesamtinib) have demonstrated activity against this mutation.^6,33,36,46,75^ A spectrum of other acquired *ROS1* resistance mutations have been described, including D2033N, L2026M, L1951R, L2086F, L2000V, S1986Y/F, L2155S, and F2004C/I/V.^46,67^

In patients with ROS1 TKI-resistant tumors harboring acquired *ROS1* resistance mutations such as G2032R, a reasonable strategy is to switch to a next-generation TKI known to cover the specific mutation(s) (Figure 2). Importantly, the landscape of mechanisms of resistance following first-line use of a next-generation ROS1 TKI such as repotrectinib remains to be determined. In an early exploratory analysis of paired baseline and post-progression ctDNA samples from TRIDENT-1, no acquired *ROS1* resistance mutations were reported among TKI-naïve patients treated with repotrectinib.^6^ Based on the experiences with third-generation EGFR and ALK TKIs, the use of more potent ROS1 TKIs upfront may result in diminished emergence of on-target resistance.^76,77^ Nevertheless, certain *ROS1* resistance mutations will remain refractory to the currently available next-generation ROS1 TKIs and are anticipated to emerge even with first-line use of next-generation agents. In particular, *ROS1* L2086F is known to be resistant to all type 1 ROS1 TKIs including repotrectinib, taletrectinib, and zidesamtinib.^7,46,78^ Here, TKI-type switching to a type 2 inhibitor like cabozantinib or a type 1 FLT3 inhibitor like gilteritinib has been identified as a potential strategy, with clinical responses to cabozantinib reported.^7,78^

#### Off-target resistance with bypass pathway activation

Various ROS1-extrinsic mechanisms of resistance have been described as drivers of ROS1 TKI-refractory disease. Broadly, these include off-target activation of signaling pathways or histologic transformation (Figure 3).

Genomic aberrations in the *MET* gene, in the form of amplifications or mutations, represent a shared off-target resistance mechanism across subsets of NSCLC including *EGFR*-mutated or *ALK*, *ROS1*, *RET* fusion-driven NSCLC. *MET* amplification has been identified after treatment with various ROS1 inhibitors including crizotinib, entrectinib, and lorlatinib.^7,68,69^ Importantly, acquired resistance driven by *MET* amplification is potentially actionable using a ROS1/MET co-inhibition strategy. Although prospective trials evaluating combinations of ROS1 and MET inhibitors have not been performed, clinical responses to crizotinib monotherapy (as an anti-ROS1/MET multikinase inhibitor) and to lorlatinib plus capmatinib combination have been reported in patients with ROS1+ NSCLC and acquired *MET* amplification.^68,79^ In patients with *EGFR*-mutated NSCLC and *MET* amplification-driven resistance to EGFR TKI, the combination of EGFR TKI plus MET TKI has demonstrated ORRs ranging 27%-67%.^80,81^ In ALK+ NSCLC with *MET* amplification-driven resistance to ALK TKI, a retrospective case series reported an ORR of 42% achieved with combined ALK and MET inhibition, concordant with efficacy seen in the prospective EGFR trials and supportive of the combination strategy.^68,82,83^

A panoply of additional off-target resistance mechanisms have been described after treatment with ROS1 TKIs, including *KRAS* mutations and amplifications, *NRAS* mutation, *NF1* loss, *MAP2K1* mutation, and activating mutations in *KIT* and *EGFR.*^7,67,70-72^ As next-generation ROS1 TKIs move into first-line, an increasing prevalence of off-target mechanisms of resistance is anticipated, underscoring the importance of defining additional bypass pathways and evaluating potential targeted therapeutic strategies.

#### Off-target resistance with histologic transformation

Although a rare occurrence in ROS1+ NSCLC, small cell transformation has been described in approximately 2% of patients with ROS1 TKI-resistant disease, with inactivation of RB1 and TP53 and loss of ROS1 fusion RNA and protein expression.^73^ This is similar to small cell transformation in *EGFR*-mutated NSCLC, where dependence on EGFR is lost. In *EGFR*-mutated NSCLC, baseline inactivation of RB1 and TP53 are associated with a 43-fold increase in the risk of small cell transformation, although the mechanisms underlying transformation are yet to be elucidated.^84,85^ Transformation from *EGFR*-mutated and ALK+ lung adenocarcinoma to squamous cell carcinoma has also been described.^86,87^ Histologic transformation, albeit infrequent, underscores the importance of obtaining a tissue biopsy at disease progression to understand disease biology and determine treatment options.

Further research is needed on the optimal treatment of small cell- or squamous cell-transformed ROS1+ lung cancer. Currently, there are no biology-directed therapeutic interventions for histology-transformed tumors, and treatments are confined to histology-specific chemotherapies and clinical trials. In patients with small cell-transformed *EGFR*-mutated lung cancer, variable degrees of clinical benefit from platinum-etoposide or taxane have been reported.^88^

#### No known targetable resistance driver

For patients without a known targetable resistance mechanism after a ROS1 TKI, the standard-of-care remains histology-specific chemotherapy. Whether to continue a CNS-penetrant ROS1 TKI when initiating chemotherapy remains an open question. In *EGFR*-mutated NSCLC, the prospective randomized COMPEL trial (NCT04765059) is examining the continuation of osimertinib versus placebo with chemotherapy after first-line osimertinib.^89^ In ALK+ NSCLC, retrospective analysis has suggested a significant benefit when a CNS-active ALK TKI is continued with chemotherapy after disease progression on next-generation ALK TKIs.^90^

Anti-PD(L)1 immune checkpoint inhibitor (ICI) monotherapy does not offer significant benefit for patients with ROS1+ NSCLC. Studies have demonstrated a modest ORR of 13%-17% with single-agent ICI.^91–93^ Furthermore, prior exposure to an ICI can augment toxicities with subsequent targeted therapies across NSCLC, and the safety impact of sequential ICI and next-generation ROS1 TKI remains to be characterized fully.^94,95^ Thus, single-agent immunotherapy is not a favored treatment strategy and should only rarely be considered when other treatment options have been exhausted, with vigilant monitoring for toxicities if patients are subsequently reintroduced to a ROS1 TKI. The role of combined chemoimmunotherapy in this patient population is not well defined. In one multi-institutional retrospective study, chemoimmunotherapy yielded objective responses in 5 of 6 patients (ORR 83%) with ROS1+ NSCLC.^93^ Randomized phase III trials exploring the question of chemoimmunotherapy versus chemotherapy alone in *EGFR*-mutated NSCLC after EGFR TKI demonstrated no substantial PFS or OS benefit (ie, KEYNOTE-789, CheckMate-722, and ORIENT-31).^96–100^ In contrast, combining chemoimmunotherapy together with an anti-angiogenic agent such as bevacizumab has yielded conflicting data in trials that largely evaluated patients with *EGFR*-mutated NSCLC (ie, IMpower150 subgroup analysis, IMpower151, ATTLAS, and ORIENT-31).^98,101–103^

In general, clinical trials should be explored for patients progressing on ROS1 TKIs with no targetable mechanisms of resistance. Antibody-drug conjugates represent an emerging therapeutic modality that has demonstrated some activity in patients with fusion oncogene-driven (including ROS1+) NSCLC, with responses observed irrespective of the mechanism of TKI resistance.^104–106^ Further development of resistance mechanism-agnostic treatment strategies for patients with ROS1 TKI-resistant NSCLC is warranted.

## Approach to non–metastatic *ROS1* fusion-positive NSCLC

Across NSCLC with actionable oncogenic drivers, targeted therapies have started to move into earlier-stage disease. Below, we review the current treatment paradigm for non–metastatic ROS1*+* NSCLC, recognizing the limitation that it is one that has been mostly informed by emerging data in *EGFR*-mutated and ALK+ NSCLC.

### Adjuvant therapy after surgical resection

The phase III ADAURA trial heralded targeted therapy in surgically resected disease, demonstrating the significantly prolonged disease-free survival (DFS; hazard ratio [HR] 0.20) and OS (HR 0.49) associated with adjuvant EGFR TKI osimertinib compared to placebo in patients with completely resected *EGFR*-mutated stage IB-IIIA NSCLC.^107,108^ Subsequently, the phase III ALINA trial demonstrated the substantial improvement in DFS (HR 0.24) achieved with adjuvant ALK TKI alectinib compared to platinum-doublet chemotherapy in patients with resected ALK+ stage IB-IIIA NSCLC, indicating that the expanded role for targeted therapy may be generalizable across biomarkers.^109^ The dramatic benefit achieved with adjuvant osimertinib and alectinib and their FDA approvals established adjuvant targeted therapy rather than ICI as standard-of-care for these disease subsets regardless of PD-L1 expression (ie, even for PD-L1 ≥
 50%), despite the genotype-agnostic approvals of atezolizumab and pembrolizumab (based on IMpower010 and KEYNOTE-091, respectively).^110,111^

Prospective data evaluating adjuvant ROS1 TKI in surgically resected ROS1+ NSCLC are lacking, and there are currently no ongoing clinical trials assessing this question. Nevertheless, extrapolating from the adjuvant TKI trials in *EGFR*/*ALK* subsets and the evidence that immunotherapy confers minimal benefit in the metastatic setting, the use of adjuvant anti-PD(L)1 ICI is not favored for resected ROS1+ NSCLC.^92^ Understanding the efficacy and long-term tolerability of next-generation ROS1 TKIs will help inform their optimal placement in the non–metastatic treatment landscape.

### Neoadjuvant or perioperative therapy in resectable cancer

For patients with resectable stage IB-IIIA and IIIB [T3, N2] NSCLC, NCCN guidelines recommend testing for PD-L1 status, *EGFR* mutation, and *ALK* rearrangement to guide neoadjuvant or perioperative systemic therapy.^17^ While numerous randomized phase III studies (eg, KEYNOTE-671, CheckMate-816, CheckMate-77T, Neotorch, and AEGEAN), have established the role of perioperative chemoimmunotherapy in resectable NSCLC, these studies have excluded patients with *EGFR*-mutant or *ALK*-rearranged NSCLC or included small numbers, limiting interpretation for patients with actionable drivers.^112–116^ Thus, the role of neoadjuvant or perioperative immunotherapy for patients with resectable ROS1+ NSCLC is unclear. The role of ROS1 TKI in the neoadjuvant or perioperative setting for patients with resectable stage IB-III disease is being explored in the NAUTIKA1 phase II trial (NCT04302025).^117^

### Consolidation therapy after concurrent chemoradiation in unresectable cancer

The standard-of-care for patients with unresectable stage III NSCLC following definitive concurrent chemoradiation is consolidation immunotherapy with durvalumab given the DFS and OS benefit demonstrated by the phase III PACIFIC study and resultant genotype-agnostic FDA and EMA approvals.^118–120^ However, among patients enrolled in PACIFIC, only 6% had a known *EGFR* mutation and other oncogenic drivers were not characterized. A post hoc PACIFIC subgroup analysis and additional retrospective studies subsequently showed that patients with *EGFR*-mutated NSCLC do not derive significant DFS or OS benefit from consolidation durvalumab.^121–124^ Furthermore, the phase III LAURA study of maintenance osimertinib versus placebo in patients with unresectable stage III *EGFR*-mutated NSCLC treated with definitive chemoradiation demonstrated a PFS benefit (OS data immature).^125^ Extrapolating to ROS1+ NSCLC, robust data to support the use of consolidation durvalumab are lacking, and exploration of a role for maintenance ROS1 TKI following concurrent chemoradiation is warranted.

## Conclusions and future directions

Over the past two decades, our understanding of the biology and successful targeting of NSCLC harboring *ROS1* fusions has progressed tremendously, transforming care for patients. Each critical advance—from the discovery of *ROS1* fusions as a biomarker in NSCLC and the approval of crizotinib as the first ROS1-targeted therapy to the characterization of resistance mechanisms and development of next-generation ROS1 inhibitors—has tangibly improved outcomes. Going forward, building upon these advances will require focused efforts to (1) refine the optimal sequencing of ROS1 TKIs and resistance landscape as next-generation inhibitors move into first-line use, (2) develop approaches to overcome off-target resistance, including mechanism-agnostic strategies, (3) employ strategies to ablate drug-tolerant persister cells and residual disease on targeted therapy to delay or prevent the emergence of drug resistance and prolong time on TKI,^126,127^ (4) move ROS1-directed therapies into the non–metastatic setting, (5) deepen our understanding of risk factors for developing ROS1+ NSCLC to inform early detection and risk reduction strategies, and (6) optimize integration of palliative and survivorship care to improve quality of life.^128–130^

The evolving landscape for the treatment of ROS1+ NSCLC is reason for hope for patients, caregivers, and clinicians, and much more hope lies ahead. We anticipate that continued research and advances in both therapeutics and early detection will improve the longevity and well-being of people living with ROS1+ lung cancer.

## Data Availability

No new data were generated or analyzed in support of this research.
